# Convenient method to improve efficiency of lymph node examination after gastrectomy with D2 lymphadenectomy for gastric cancer

**DOI:** 10.1186/s12876-023-03061-2

**Published:** 2023-12-06

**Authors:** Hanting Xiang, Zhebin Dong, Hengmiao Wu, Yicheng He, Zhengwei Chen, Sangsang Chen, Weiming Yu, Chao Liang

**Affiliations:** Department of General Surgery, the Affiliated Lihuili Hospital, Ningbo University, Ningbo, 315000 People’s Republic of China

**Keywords:** Gastric cancer, Gastrectomy, Lymph node examination

## Abstract

**Background:**

The D2 procedure has been accepted as the standard treatment for advanced gastric cancer (GC) in East Asia. Determination of the number of lymph nodes (LNs) after gastrectomy may influence the pathological stage assessment of lymph node metastasis, significantly influencing prognostic evaluations and formulation of chemotherapy regimens.

**Methods:**

Between January 2020 and January 2022, the medical files of 312 patients with clinical stage T0-4aN0-3M0 gastric cancer were reviewed retrospectively, and the patients were assigned to the normal group (lymph nodes were examined roughly), manual group (lymph nodes were manually examined meticulously), and device group (lymph nodes were examined by device). The clinical and pathologic characteristics, number of lymph nodes harvested, and the time required for lymph node examination was compared.

**Results:**

A total of 312 gastric cancer patients (mean age 65.8 ± 10.3 years, 85 females and 227 males) underwent gastrectomy with curative intent at our department. Sex, age, body mass index (BMI), tumor size, clinical TNM stage, and pathologic TNM stage in the three groups showed no statistically significant differences (P > 0.05). The mean number of harvested lymph nodes in the normal, manual, and device group was 24.2, 36.6 and 35.2, respectively, which showed significant differences (P < 0.0001). The mean number of positive lymph nodes in the normal, manual, and device group was 3.5, 3.9 and 3.9, respectively (P = 0.99). The mean time consumption in device group was 15 min while the time consumption in manual group was 52.3 min, which showed a significant difference (P < 0.0001).

**Conclusion:**

This improved lymph node examination method offers a simple approach that is worth promoting, and it can improve the number of harvested lymph nodes efficiently.

## Background

Gastric cancer (GC) is the fourth leading cause of cancer-related death and fifth in terms of incidence globally, affecting more than 400,000 patients per year in China [1]. According to the Chinese GC diagnosis and treatment guidelines, the current standard treatment for advanced GC patients is D2 gastrectomy followed by adjuvant chemotherapy [2]. Unfortunately, more than 80% of Chinese patients are diagnosed at advanced stages, and surgery with curative intent requires extended lymphadenectomy [3]. Several studies have shown that the postoperative survival of GC patients is correlated with the number of lymph nodes (LNs) harvested and LN metastasis [4, 5]. Therefore, examination of 16 or more regional LNs for N status determination is recommended by Japanese Gastric Cancer Association [6]. The Surveillance, Epidemiology, and End Results (SEER)-based cut-off point analysis also showed the greatest difference in survival between 10 LNs examined, but the difference was significant at cut-off points up to 40 LNs [7]. The number of harvested LNs is an independent factor affecting prognosis even for patients whose LNs are all negative [4, 8].

Thus, accurate diagnosis of the N stage is important to judge the prognosis of patients and to administer postoperative adjuvant therapy [4, 5, 9, 10]. However, according to the research by Sano et al., the mean number of LNs harvested by some large centers in China was only 24.8, while the corresponding numbers in Japan and Korea were 39.4 and 33.0, respectively [11]. Thus, the LN harvested number of GC specimens needs to be urgently improved in China. In a study on standardized D2 LN dissection, the number of LNs harvested was mainly affected by the method of examination [12]. Therefore, optimization of the LN examination method has become a key point to improve the number of LN harvested after gastrectomy in China.

**Fig. 1:**
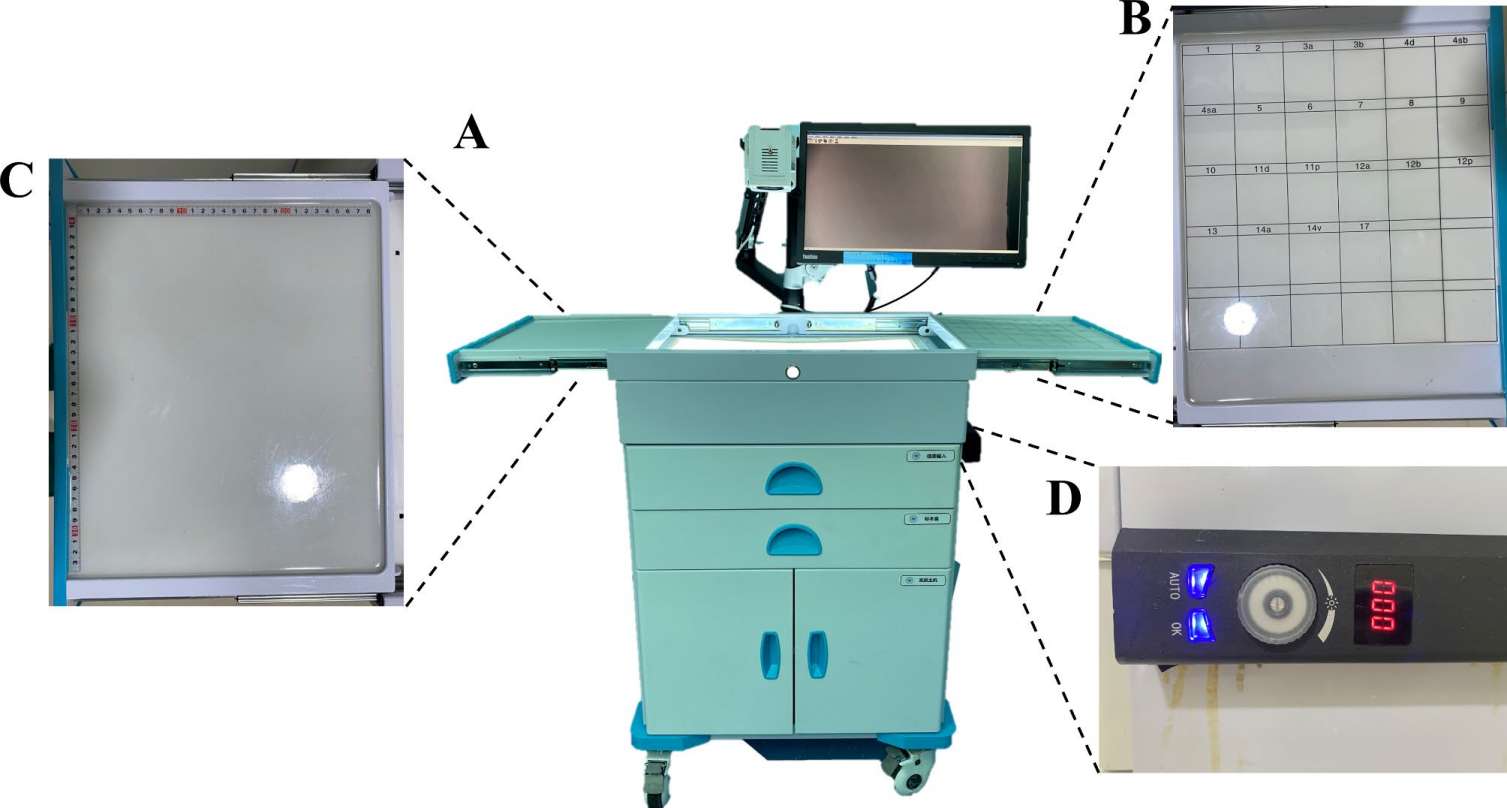
Electronic lymph node examination device. **A**: Holistic view of the electronic lymph node examination device. **B**: Lymph node grouping table. **C**: Specimen measurement table. **D**: Brightness adjustment knob

Currently, LN examination in most medical centers in China is conducted by surgeons who perform only a rudimentary processing of the specimen, after which it is then passed on to pathologists for further LN examination. Limited by the expertise of the pathologists, number of harvested LNs often unsatisfactory. LNs in formalin-treated specimens are also more difficult to detect, often result in crude portioning of the LN in pathological reports. Moreover, the number of harvested LNs often depends on the interest level of the pathologists. Consequently, the greatest number of detected LNs was unsatisfactory. As a result, Chinese experts have published a consensus [13] to standardize the surgical processing procedure of GC specimens. However, while this method can greatly improve the harvested number of LNs, it requires a significant amount of time and manpower, thereby adding further burden to an already demanding clinical workload. Due to the demanding nature of healthcare work in China, the vast majority of surgeons still adopt the initial approach to LN examination rather than the “gold standard” proposed by the consensus. To improve the efficiency of LN examination by surgeons, we have developed an electronic device for LN examination (Fig. 1A).

**Fig. 2:**
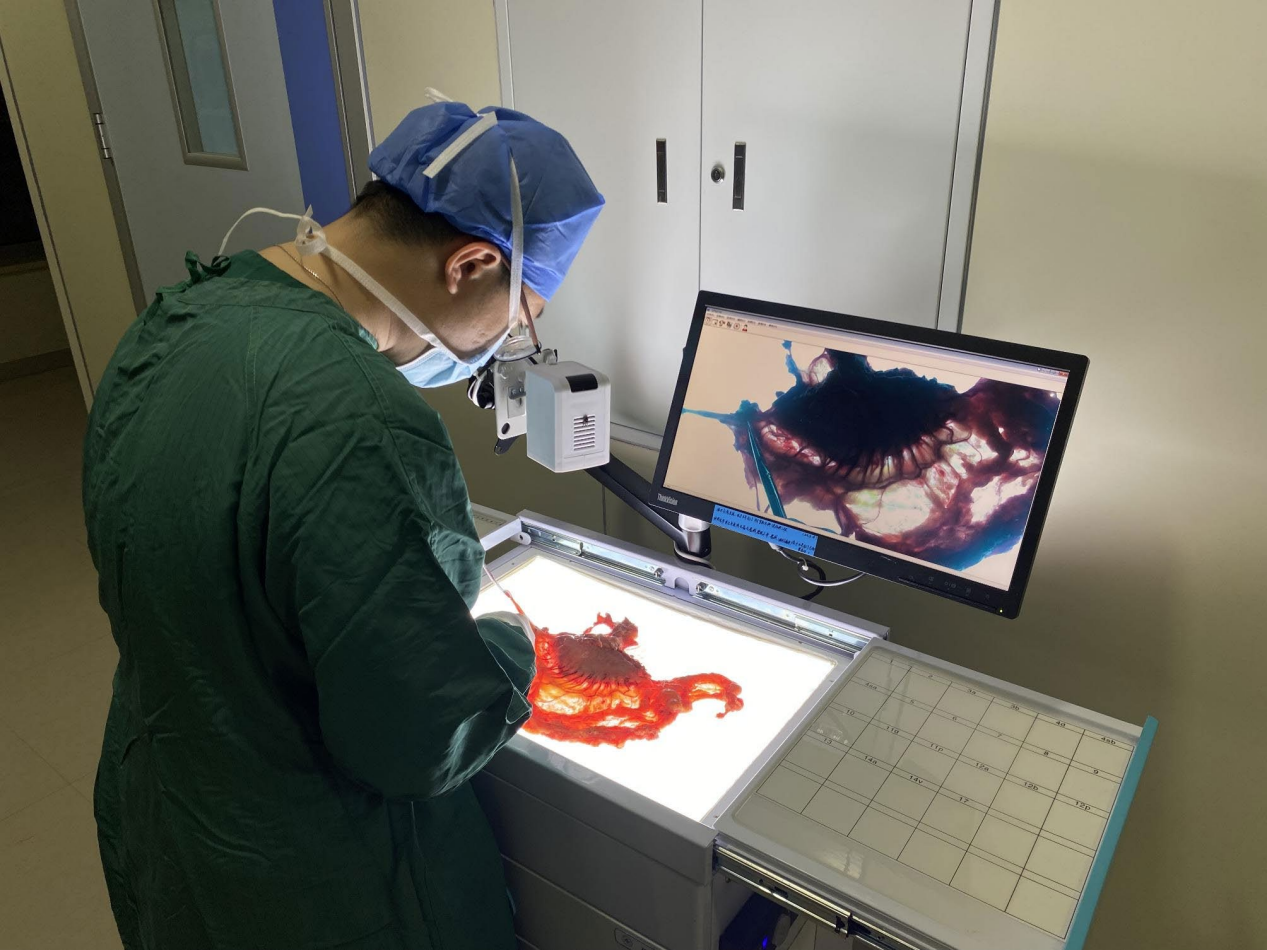
Dedicated lymph node examination area in the operating room

This is an integrated device based on LN examination, primarily consisting of an imaging system, a display monitor, a examination table, a LN grouping table (Fig. 1B), and a specimen measurement table (Fig. 1C). The imaging system can record or capture videos or photographs of the specimens at a resolution of 2K. After being connected to a network, the data can be uploaded to a cloud platform for easy access and viewing. A backlighting device with adjustable brightness (Brightness adjustment range: 0-4000 cd/m^2^, color temperature: 8000 K) is installed underneath the LN examination table, providing clear LN visual guidance for surgeons (Fig. 1D). To reduce errors in LN grouping records, the sorted LNs can be placed in the compartments of the LN grouping table according to their respective groups. In addition, a rough ruler is placed on the specimen measurement table, and finely calibrated rulers are stored in the drawer below to meet the varying specimen observation needs of surgeons. Besides, a dedicated LN sorting area have been set up in the operating room, equipped with specialized instruments and surgical towels (Fig. 2).

In the currently study, we compared the number of LNs harvested and the time consumed by traditional LN examination, the “gold standard” for LN examination proposed by the consensus, and the electronic device-based LN examination. The results indicated that the utilization of the electronic device for LN examination not only effectively increased the number of harvested LNs but also resulted in significant time savings compared to LN examination proposed by the consensus. Thus, this method holds promise for widespread adoption.

## Methods

### Patients

Ethics committee permission for accessing patients’ data was obtained prior to the initiation of this study. We included patients aged between 20 and 90 years who were diagnosed with GC, met the indications for distal or total gastrectomy with D2 lymphadenectomy; and showed no distant metastasis on intraoperative examination. However, we excluded patients who had undergone proximal gastrectomy or pylorus-preserving gastrectomy; had severe organ dysfunction that precluded surgery; had a history of unstable angina, myocardial infarction, or cerebrovascular accident within the past 6 months; had incomplete pathological material; had undergone palliative excision; showed a large tumor or severe local infiltration on preoperative examinations; or showed distant metastasis on preoperative examinations.

According to the criteria above, the medical files of patients who presented to our department with a diagnosis of GC and underwent gastric resection with curative intent at the Affiliated Lihuili Hospital, Ningbo University, between January 2020 and January 2022 were retrospectively reviewed. A total of 312 patients were divided into normal group (LNs were examined roughly), manual group (LNs were examined meticulously by hand), and device group (LNs were examined by device).

### Method of examination

All specimens were processed by experienced surgeons with no conflict of interest, and then sent to Ningbo pathology center for examination by pathologist. The LNs were examined through the following method:

Normal group: Surgeons roughly divides the LNs into right cardia, left cardia, lesser curvature, greater curvature, suprapyloric, subpyloric before the specimens fixed with formalin, and then sent to the pathologist for pathological diagnosis.

**Fig. 3:**
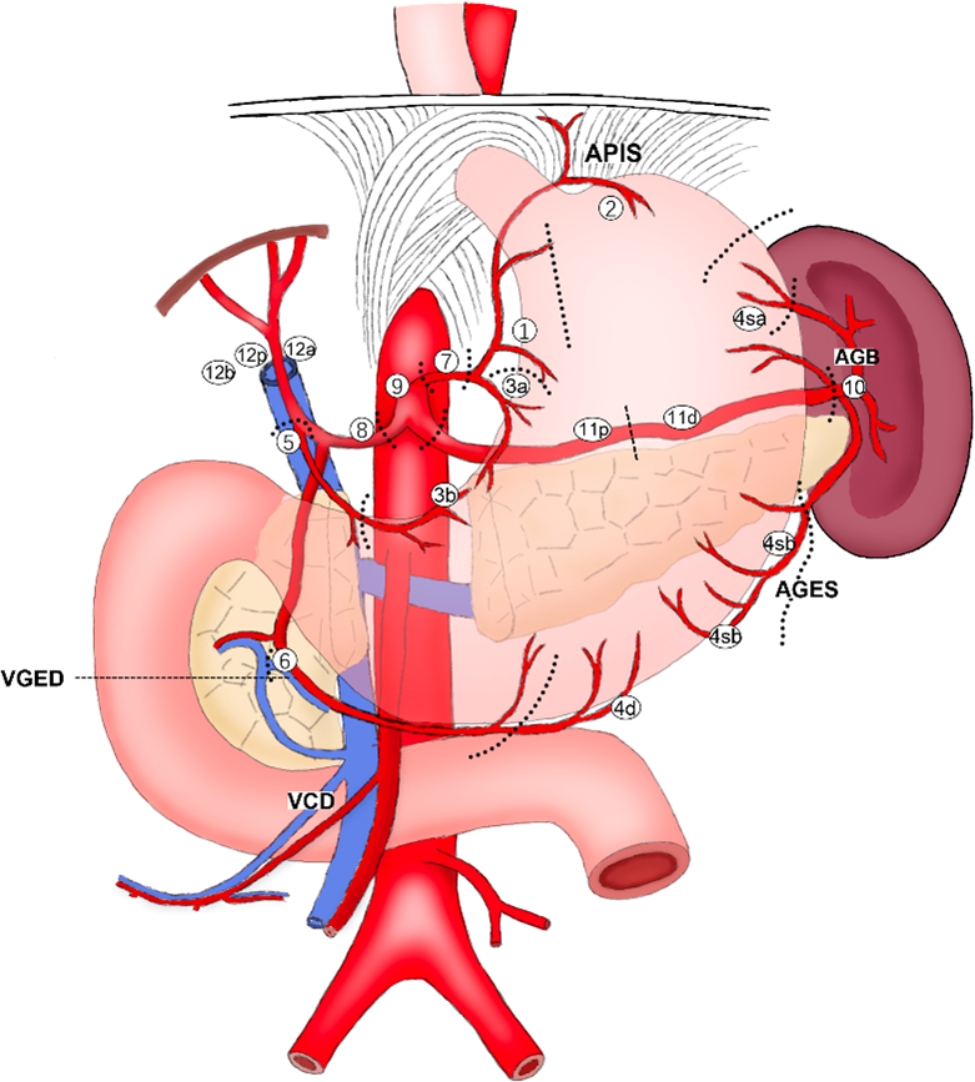
Schematic diagram of lymph node grouping. Abbreviations: AGB: Short gastric artery; AGES: Left gastroepiploic artery; APIS: Inferior phrenic artery; VCD: Right colonic vein; VGED: Right gastroepiploic vein

Manual group: The specimens were manually and standardly managed based on the Chinese experts’ consensus [13], and then sent to the pathologist for pathological diagnosis after fixed with formalin. The schematic diagram for LN grouping is shown in Fig. 3.

**Fig. 4:**
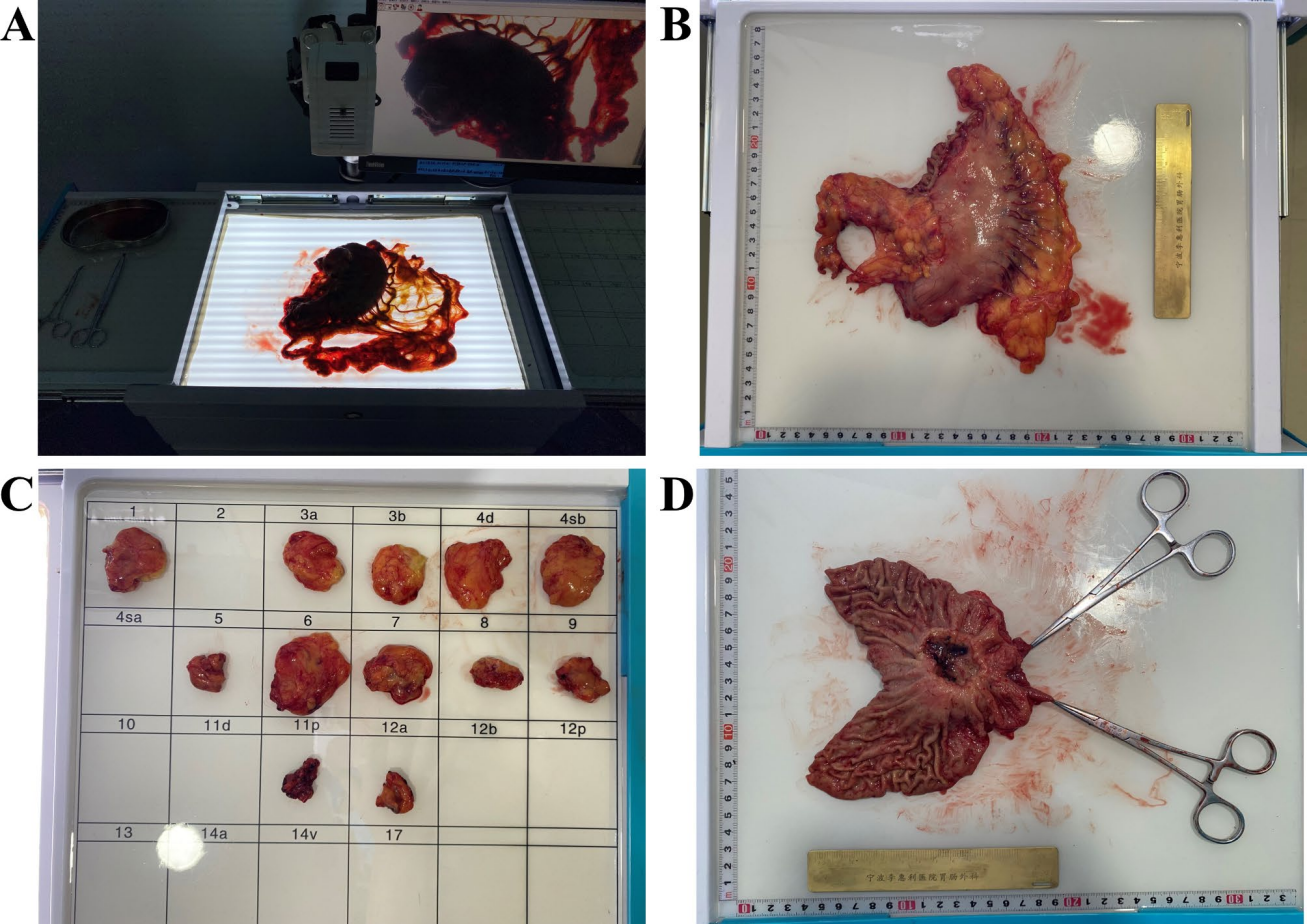
Lymph node examination process based on the electronic lymph node examination device. **A**: Place the specimen in its anatomical position according to its location in the body. **B**: Measure the size of the specimen. **C**: Place the harvested lymph nodes in numerical order. **D**: Measure the size of the tumor

Device group: The specimens were placed on the backlit table in accordance with the physiological and anatomical distribution and photography after cleaning the mucus and blood from the specimen with gauze or bibulous paper (Fig. 4A). Pulling the broken end of a vessel that was marked intraoperatively and fully unfolding the gastric omentum. Subsequently, adjust the brightness knob to achieve the appropriate backlight brightness. Photographing the specimens through the imaging system and upload them to the database. Additionally, use the ruler to measure the size of the specimens and record it in the database (Fig. 4B). Processing the specimens in accordance with the Chinese experts’ consensus and place the sorted LNs into the corresponding compartments on the LN grouping table (Fig. 4C). Then measure the tumor diameter after cutting open the stomach along the lesser curvature (Fig. 4D). Lastly, the specimens were fixed with formalin and sent to the pathologist for pathological diagnosis.

The sample size was determined using a sample size calculation formula for independent sample mean comparison in a previous study [14, 15]. The extent of gastrectomy and D2 LN dissection was based on the Japanese GC treatment guidelines [6]. All procedures were performed under the following standardized principles, as mentioned, by surgeons who had performed at least 500 gastrectomies with D2 lymphadenectomy using open and laparoscopic approaches.

### Statistical analyses

SAS 9.3 software and GraphPad Prism v9.4.0 software were used for statistical analyses. The data are presented as the mean and standard deviation for continuous variables and as numbers for categorical variables. The differences between groups were calculated by using the Kruskal–Wallis H test when the data were not normally distributed, while analysis of variance (ANOVA) was used when the data were normally distributed. In addition, an unpaired t-test and Chi-square test was also used to compare differences between groups. All P values were two-sided, and a P value less than 0.05 indicated significant difference.

## Results

Between January 2020 and January 2022, a total of 312 GC patients (mean age 65.8 ± 10.3 years, 85 females and 227 males) underwent gastrectomy (111 laparoscopic surgery, 201 open surgery) with curative intent in our department. Clinical TNM stage was similar in normal, manual and device group (P = 0.91).

The clinical and pathologic characteristics of the three groups are provided in Table 1. Baseline characteristics, including sex, age, and body mass index (BMI) were similar in the three groups (P > 0.05). Tumor distribution (P = 0.88), histologic type (P = 0.96), degree of differentiation (P = 0.18), and pathologic tumor stage (P = 0.75) were also comparable in the three groups and showed no significant differences.

The surgical outcomes are presented in Table 2. Based on the location of the primary tumor and the desired specimens’ tumor-free borders, distal gastrectomy was performed for 71 patients (68.3%) in the normal group, 77 patients in the manual group (74.0%), and 75 patients (72.1%) in the device group. On the other hand, 33 patients (31.7%) in the normal group, 27 patients (26.0%) in the manual group, and 29 patients (27.9%) in the device group received total gastrectomy (P = 0.64). In all cases, extended (D2) LN dissection was performed. On the basis of the results of the statistical analysis, the mean surgical time in the normal, manual, and device groups was 197.5, 203.5 and 202.0 min, respectively (P = 0.51).

The mean time consumption of LNs examination in the normal and device groups was 52.3 and 15 min, respectively, which were significantly different (P < 0.0001). Furthermore, the mean number of LNs harvested in the normal group was 24.2 less than that in the manual and device group, which showed a statistically significant difference (24.2 vs. 36.6 vs. 35.2, p < 0.0001). However, the mean number of harvested LNs in the manual and device group was similar (P = 0.33). The mean number of positive LNs in the normal, manual, and device groups was 3.5, 4.1 and 3.9, respectively. Although these findings showed was a trend, statistical significance was not reached for the three groups (P > 0.50).

**Fig. 5:**
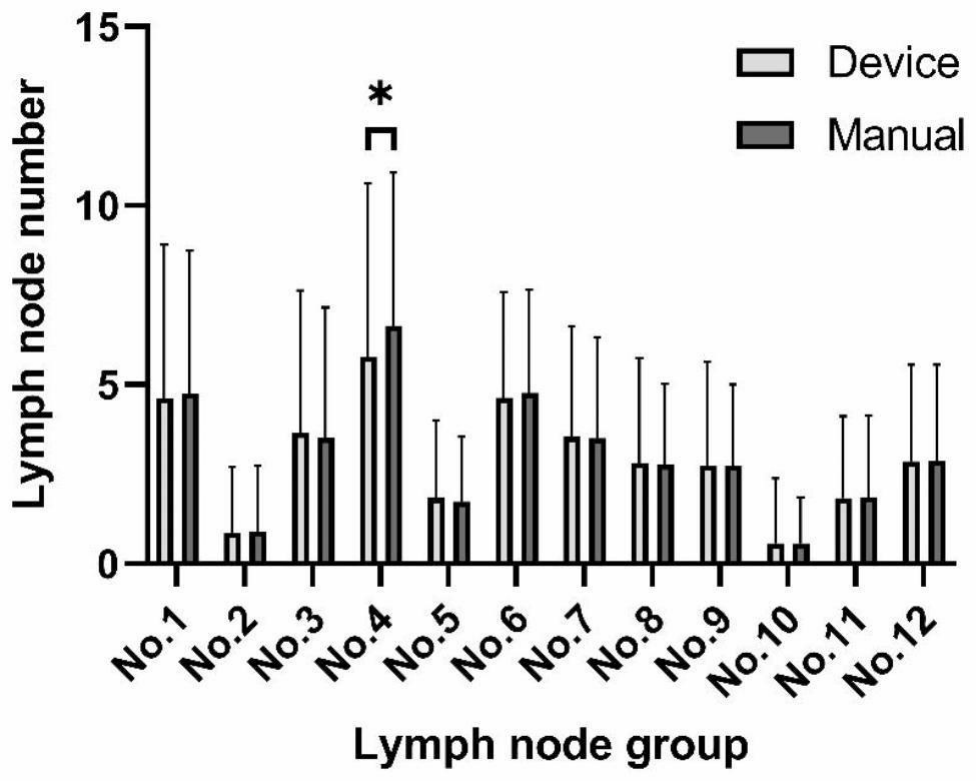
Harvested lymph node number of each group in manual and device group

In addition, we compared the number of LNs in each subgroup of the manual and device group. The number of each LN group in manual and device group was showed in Fig. 5. The number of LNs was similar in all groups of both manual and device groups except for the fourth group (5.8 vs. 6.6, P = 0.046).

**Table 1:** Patient clinical and pathologic Characteristics

Characteristic	Normal					Manual					Device				P
	No.	%	Mean	SD		No.	%	Mean	SD		No.	%	Mean	SD	
Sex															0.60
Male	72	69.2				77	74				78	75			
Female	32	30.8				27	26				26	25			
Age, years			65.2	11.7				65.5	9.8				66.8	9.4	0.59
BMI, kg/m^2^			22.6	3.5				22.3	3.1				22.1	3.1	0.55
Tumor size, cm			3.9	2.3				4.1	2.5				4.3	2.7	0.79
Histology															0.48
Adenocarcinoma	95	91.3				98	94.2				99	95.2			
Signet-ring cell carcinoma	5	4.8				6	5.8				4	3.8			
Neuroendocrine tumor	1	1.0				0	0.0				1	1.0			
Low adhesion carcinoma	3	2.9				0	0.0				0	0.0			
Differentiated degree															0.16
Low	81	77.9				72	69.2				69	66.3			
Medium	21	20.2				29	27.9				32	30.8			
High	2	1.9				3	2.9				3	2.9			
Tumor distribution															0.88
Upper third	13	12.5				9	8.7				11		10.6		
Middle third	29	27.9				32	30.8				33		31.7		
Lower third	62	59.6				63	60.6				60		57.7		
Pathologic T stage															0.66
<T2	36	34.6				29	27.9				33	31.7			
T2-4a	68	65.4				75	72.1				71	68.3			
Pathologic N stage															0.97
N0	52	50.0				54	51.9				52	50.0			
N1	14	13.5				9	8.7				11	10.6			
N2	15	14.4				12	11.5				16	15.4			
N3	23	22.1				29	27.9				25	24.0			
Pathologic TNM stage															0.97
<IB	31	29.8				26	25.0				28	26.9			
IB	9	8.7				11	10.6				9	8.7			
IIA	12	11.5				12	11.5				12	11.5			
IIB	13	12.5				16	15.4				17	16.3			
IIIA	12	11.5				12	11.5				12	11.5			
IIIB	21	20.2				26	25.0				24	23.1			
IIIC	6	5.8				1	1.0				2	1.9			
Clinical T stage															0.80
<T2	36	34.6				34	32.7				33	30.5			
T2-4a	68	64.4				70	67.3				71	64.7			
Clinical N stage															0.51
N0	50	48.1				55	52.9				54	49.9			
N1	17	16.3				20	19.2				19	17.6			
N2	17	16.3				16	15.4				18	16.6			
N3	20	19.2				13	12.5				13	12.0			
Clinical TNM stage															0.91
<IIA	39	37.5				40	38.5				39	36.1			
IIA	4	3.8				3	2.9				4	3.7			
IIB	11	10.6				16	15.4				15	13.9			
III	50	48.1				45	43.3				46	42.5			

NOTE: Tumor stage according to the Chinese Society of Clinical Oncology Clinical guidelines(2021).

Abbreviations: BMI: Body mass index; SD: Standard deviation.

**Table 2:** Surgical outcomes

Outcome	Normal					Manual					Device				P
	No.	%	Mean	SD		No.	%	Mean	SD		No.	%	Mean	SD	
Gastrectomy															0.64
Distal	71	68.3				77	74.0				75	72.1			
Total	33	31.7				27	26.0				29	27.9			
Surgical time, minutes			197.5	26.7				203.5	33.0				202.0	23.8	0.51
Number of harvested LNs			24.2	7.6				36.6	10.5				35.2	12.0	<0.0001
Number of positive LNs			3.5	5.1				4.1	6.5				3.9	6.6	0.99
Time consumption of LNs examination								52.3	4.3				15	3.1	<0.0001

Abbreviations: SD, standard deviation.

## Discussion

Supporting data from a substantial number of publications have highlighted the importance of the LN ratio, namely the ratio of positive to resected LNs, in the prognosis of gastric, pancreatic, and colorectal cancer [4, 16–18]. The short and long-term prognoses of GC and generally all gastrointestinal epithelial malignancies are determined after a careful TNM staging based on information derived from both pathological and radiological assessments. The nodal status appears to be of the utmost importance since it usually dictates the need for subsequent adjuvant therapies [4, 16]. The greater the number of LNs examined, the better the resulting post-gastrectomy survival in patients with T1-3N0-1 GC. Regardless of the underlying mechanisms that influence the impact of LN counts on survival, the results call for attention to the analysis of the total LN number as an important and powerful qualifier of staging information and survival prediction for GC in future clinical trials [7].

Surgeons in our center took note of these issues and invented this electronic LN detection device to help surgeons examine LNs faster and more efficiently. Given that the greater omentum has some level of translucency, we attempted to add a backlighting system beneath the specimen. With the backlight located at the base of the device, the blood vessels and LNs of the specimen can be vividly and distinctly visualized, which would assist surgeons in processing specimens and examine LNs more efficiently. In our center’s research, preliminarily trained surgeons can sort a significantly higher quantity of LNs in just 15 min compared to the normal procedure. In addition, the device is equipped with designated areas for different groups of examined LNs, eliminating the need for additional personnel to document the examined LNs groups and significantly reducing the likelihood of errors in group classification. Subsequent benefits such as a greater number of positive LNs, more specific TNM staging, and potentially better prognosis were self-evident. Moreover, this electronic device has the capability to capture, store, and share images, providing immense convenience to surgeons for archiving and reviewing patient-specific data. Further improvements in specimen processing can not only enhance accurate staging of GC after surgery but also aid in developing supplementary treatment strategies and estimating prognosis, thereby enhancing the overall prognosis for patients with GC.

In the training of new examination personnel, we mainly use three steps: (1) Theoretical foundation: New trainees first learn and familiarize themselves with gastric anatomy, and then receive theoretical lectures on LN examination and the device from experienced surgeons. This helps trainees gain a systematic and comprehensive understanding of gastric LN stations and identification. (2) Identification and corresponding specimens in vivo and in vitro: Trainees will observe and learn about various GC surgical procedures from the operating room to gain an understanding of how surgeons separate tissues corresponding to each LN station and how they disconnect the corresponding blood vessels. This allows trainees to better understand the adjacent relationships of various tissue parts and the sources of vascular clips after the specimens are removed, and to better correspond the in vitro status of specimens with their in vivo state. Furthermore, during the observation of surgical procedures, it is sometimes possible to witness the surgeon isolating and excising certain noticeably enlarged LNs, which can enhance the trainees’ ability to discern the morphology and texture of LNs. (3) Ex vivo LN examination: After completing the aforementioned stages of training, the trainees will perform LN examination under the guidance of experienced surgeons. Furthermore, to maintain a consistently high quality of LN examination in GC specimens, each trainee is required to report the number of LNs harvested in each GC specimen. The surgeon will review the final pathological results at the time of the patient’s discharge and compare them with the reported number of harvested by the trainee to identify any significant discrepancies. A root cause analysis is then performed for any variable values detected.

On the other hand, improving surgical quality also contributes significantly to increasing the number of detected LNs. Currently, laparoscopic surgery is widely adopted among GC surgeons. However, due to the increased complexity of laparoscopic procedures, there have been concerns from an oncological perspective about the safety and effectiveness of laparoscopy, especially for advanced GC cases that require extensive D2 LN dissection. Most research reports suggest that there is no significant difference in LN retrieval between laparoscopic and open surgery [19]. Given that another advantage of laparoscopic surgery is the lower incidence of wound complications, performing laparoscopic surgery in experienced centers is likely to be more recommended [20]. Of course, it’s essential to ensure that the surgeon has the necessary qualifications for a thorough LN dissection before the procedure is carried out.

We found that the number of LNs detected in the fourth group was smaller in the device group than in the manual group while the total number of detected LNs was similar in device group and manual group. This may be related to the larger distribution of LNs in the fourth group, which was divided into three groups: 4sa, 4sb, and 4d. A larger scope of inspection may result in a larger number of detections and ultimately lead to statistical differences in the number of detections.

In this study, we demonstrated a new LN detection method based on an electronic LN detection device. This method helped surgeons detect LNs quickly and efficiently after gastrectomy. However, this study still had several limitations. The retrospective nature of the study may have led to selection bias, especially for patients undergoing palliative excision, since these cases tend to be associated with more LN metastases, which are easier to detect. Moreover, all patients were included from only one hospital, and the results may have been influenced by hospital-specific practices. Therefore, long-term follow-up and randomized controlled trials (RCTs) need to be implemented in the future. Multiple-center clinical trials are also needed for further studies in the future.

At present, identification of LNs in postoperative GC specimens is primarily dependent on the surgeon’s vision and touch. However, with the application of intraoperative ultrasonography, the use of carbon nanoparticles and indocyanine green dye in LN tracing of GC also offers great application prospects [21–23]. The introduction of precision medicine concepts in LN tracing methods will make these examinations simpler and more convenient. Thus, we look forward to the development of more accurate and effective new tracers to achieve higher GC treatment effects.

## Conclusion

We developed a novel LN detection method based on an electronic LN detecting device that can effectively improve the number of LNs detected. Notably, detection of more LNs can help avoid “inappropriate understaging” of the disease and potentially improve post-gastrectomy survival. In conclusion, this is a new improved LN detection method that is worth promoting, and this LN detection method can be expected to become more efficient with the development of novel LN tracers in the future.

## Data Availability

The datasets used and analyzed during the current study are available from the corresponding author on reasonable request.

## List of abbreviations

GC: Gastric cancer
LN: Lymph node
AGB: Short gastric artery
AGES: Left gastroepiploic artery
APIS: Inferior phrenic artery
VCD: Right colonic vein
VGED: Right gastroepiploic vein
BMI: Body mass index
SD: Standard deviation
